# Supplementary figures and images for: Genome-Wide Characterization of High-Affinity Nitrate Transporter 2 (NRT2) Gene Family in Brassica napus
Source: Int J Mol Sci. 2022 Apr 29;23(9):4965. doi: 10.3390/ijms23094965 (PMC9104966; doi:10.3390/ijms23094965)

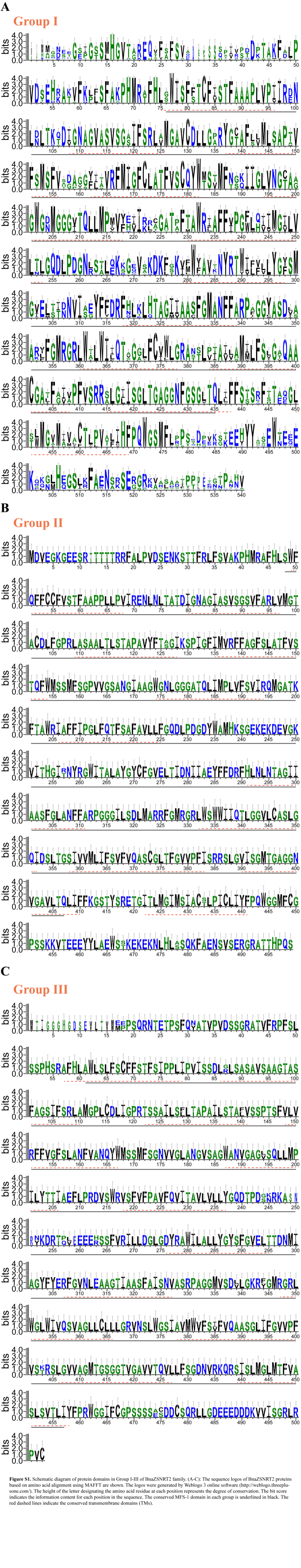

Supplement: Supplementary file 1 [file ijms-23-04965-s001.zip › ijms-1670908-supplementary/Supplementary Materials/Figure S1.pdf]
